# Supplementary material for: The past century of coral bleaching in the Saudi Arabian central Red Sea
Source: PeerJ. 2020 Oct 23;8:e10200. doi: 10.7717/peerj.10200 (PMC7587059; doi:10.7717/peerj.10200)
Supplement: Supplemental Information 1 — All cores were collected from colonies of the genus Porites. Depth indicates the water depth of the top of each colony. The oldest band indicates the age of the bottom-most annual band of each core. [file peerj-08-10200-s001.pdf]

Table S1. Coral core locations, depths, oldest counted bands, and any stress bands observed. All cores were collected from colonies of the genus *Porites*. Depth indicates the water depth of the top of each colony. The oldest band indicates the age of the bottom-most annual band of each core.

ID	Reef	Latitude (°N)	Longitude (°E)	Depth (m)	Oldest band	Stress bands
K01	Abu Shosha	22.30435	39.047	2	1964	1998, 2010
K02	Abu Shosha	22.30435	39.047	4	1986	2010
K03	Rose Reef	22.31032	38.88578	3	1996	-
K04	Shi'b Nazar	22.32012	38.85533	10	1968	-
K05	Shi'b Nazar	22.3288	38.85845	1	2000	-
K06	Cement Wreck	22.3916	38.85392	5	1934	1948, 1978
K07	Cement Wreck	22.3916	38.85392	4	1967	-
K08	Piper's Pinnacle	22.39198	38.92038	11	1944	-
K09	Al Mtarbj	22.4319	38.94892	3	1985	2010
K10	Al Mtarbj	22.4319	38.94892	2	1979	-
K11	Qita Al-Kirsh (Shark Reef)	22.42813	38.9962	6	1994	-
K12	Qita Al-Kirsh (Shark Reef)	22.42813	38.9962	6	2010	-
K13	Abu Shosha	22.30414	39.04633	4	1917	1931, 1982, 1998, 2010, 2015
K14	Abu Shosha	22.30414	39.04633	4	1934	1978, 1982, 1998
K15	Abu Shosha	22.30414	39.04633	4	1968	-
K16	Abu Shosha	22.30414	39.04633	4	1970	1998
K19	Abu Shosha	22.30487	39.04686	3	1966	1998, 2010
K20	Abu Shosha	22.30487	39.04686	2	1936	1998, 2010
K22	Abu Shosha	22.3056	39.04758	3	1970	1982, 1998, 2010
K23	Abu Shosha	22.3056	39.04758	3.5	1930	2010
K24	Abu Shosha	22.3056	39.04758	3.5	1970	1998, 2010
K25	Cement Wreck	22.3873	38.8548	11	1908	-
